# Combined serum IL-6, C-reactive protein, and cortisol may distinguish patients with anhedonia in major depressive disorder

**DOI:** 10.3389/fnmol.2022.935031

**Published:** 2022-08-24

**Authors:** Yinghui Li, Yingying Yue, Suzhen Chen, Wenhao Jiang, Zhi Xu, Gang Chen, Zixin Zhu, Liangliang Tan, Yonggui Yuan

**Affiliations:** ^1^Nanjing Medical University, Nanjing, China; ^2^Department of Psychosomatics and Psychiatry, ZhongDa Hospital, School of Medicine, Southeast University, Nanjing, China

**Keywords:** depression, anhedonia, cytokines, C-reactive protein, cortisol

## Abstract

Neuroinflammation and anhedonia in major depressive disorder (MDD) are closely connected, though the exact mechanism is unclear. This study aimed to investigate the relationships between cytokines, C-reactive protein (CRP), cortisol, and anhedonia, revealing the potential predictive value in identifying anhedonic MDD. In total, 66 patients with MDD (29 with anhedonia and 37 without anhedonia) and 66 healthy controls (HCs) were included. The severity of depression and anhedonia was evaluated using the Hamilton Rating Scale for Depression-24 (HAMD-24) and Snaith-Hamilton Pleasure Scale (SHAPS), respectively. Serum cytokines were measured using flow cytofluorometric kits, while CRP and cortisol were measured using enzyme-linked immunosorbent assay kits. We found higher serum levels of interleukin-2 (IL-2), IL-6, and cortisol in MDD than in HC where anhedonic MDD was highest. CRP and IL-6 were positively associated with anhedonia, and cortisol levels were related to both anhedonia and depression. A combination of IL-6, CRP, and cortisol had optimal predictive value for distinguishing anhedonic MDD. Anhedonic MDD has unique neuroendocrine-immune characteristics compared with those without anhedonia. The combination of IL-6, CRP, and cortisol might be an early marker to distinguish anhedonic MDD.

## Introduction

Anhedonia, the inability to experience pleasure, is one of the two core symptoms of major depressive disorder (MDD). Approximately 70% of individuals with MDD underwent anhedonic symptoms (Cao et al., 2019), and anhedonia often worse the clinical performance of MDD. Specifically, the associations between anhedonia, impaired psychosocial functioning, poorer disease prognosis, and unsatisfactory curative outcomes have been reported (Vinckier et al., 2017; McIntyre et al., 2021). In addition, anhedonia frequently persists during inter-episodic periods in MDD (Rodrigues et al., 2020). Finally, evidence suggests that anhedonia is significantly associated with suicide ideation and suicide attempts in patients with MDD (Yang et al., 2020; Ducasse et al., 2021).

Growing literature implicates that immune system dysfunction may perform a salient role in the pathophysiology of anhedonia (Pan et al., 2017; Rengasamy et al., 2021). The relationships between anhedonic symptoms and increased levels of inflammatory cytokines have received much more attention (Tang et al., 2021). In adolescents, anhedonia but not depressive symptom was associated with tumor necrosis factor-α (TNF-α), interleukin-2 (IL-2), IL-4, IL-6, IL-10, and IL-17A (Freed et al., 2019). Adolescent MDD also showed that higher levels of baseline TNF-α related positively to anhedonia longitudinally (Rengasamy et al., 2021). Similar studies on anhedonia and cytokines in adult MDD are relatively limited. A recent study dividing patients with adult MDD into anhedonic and nonanhedonic groups found increased levels of IL-6 in anhedonic MDD. However, the study did not exhibit a significant relationship between cytokines and anhedonic symptoms (Tang et al., 2021), which was inconsistent with the results in adolescents. Moreover, the cytokines involved in that study were just three (TNF-α, IL-6, and IL-10). Therefore, further studies are required to expand the number of cytokines and add other important inflammatory markers, such as C-reactive protein (CRP), to investigate the relationship between inflammation and anhedonia in patients with adult MDD.

C-reactive protein is a product of acute inflammatory response, which is mainly induced by hepatocytes to inflammatory cytokines, especially IL-6 (Felger et al., 2020). Increased CRP levels are related to the severity of depression and poor treatment response (Köhler-Forsberg et al., 2017; Jha et al., 2019). Functional magnetic resonance imaging (fMRI) studies show correlations between CRP and brain regions involved in reward circuits corresponding to anhedonia (Felger et al., 2016; Goldsmith et al., 2020; Liu et al., 2020). However, the direct study of CRP and anhedonic symptoms remains limited. The only one early research in this scope including bipolar depression, unipolar depression, and anxiety disorders (Loas et al., 2016) reported no significant findings.

Studies have shown a link between neuroendocrinology and immunity in patients with MDD (Woelfer et al., 2019). Furthermore, it is well-established that the functional changes of the hypothalamus pituitary adrenal (HPA) axis occur in patients with MDD (Alenko et al., 2020). Cortisol was closely associated with the severity of depressive symptoms as a prospective predictor of MDD diagnosis (Islam et al., 2018). Additionally, men participants showed that more severe anhedonia was substantially associated with higher cortisol levels (Cunningham et al., 2021). Although evidence has shown that increased cytokines can result in the activation of the HPA axis leading to the release of cortisol (Brymer et al., 2019; Lee et al., 2020), few studies have presented a complete pathway of inflammation, cortisol, and anhedonia simultaneously in MDD. We extended to draw a whole map to include cytokines, CRP, and cortisol along with anhedonia.

To date, no biomarker-based diagnostic panel is available. A particular cytokine and CRP were not effective predictors of distinguishing between depressed and nondepressed individuals (Del Giudice and Gangestad, 2018; Yang et al., 2018; Ting et al., 2020). Since the complex underlying pathogenesis of MDD, such as the dysfunction of the HPA axis, inflammation, and immune system alterations (Jentsch et al., 2015), the combination of multiple biological factors with discriminating effects may improve the accuracy of prediction (Xu et al., 2013; van Buel et al., 2019). To the best of our knowledge, no studies have combined cytokine, CRP, and cortisol as an indicator to distinguish MDD with anhedonia from those without anhedonia. Early differentiation of MDD with or without anhedonia can help clinicians with precision medicine achieve good treatment outcomes.

To understand the effect of CRP, cytokines, and cortisol in the pathogenesis of anhedonia in MDD, this study first analyzed and compared serum cortisol, CRP, and cytokines, including TNF-α, interferon-γ (IFN-γ), IL-2, IL-4, IL-6, IL-10, and IL-17A between patients with MDD and healthy controls (HCs). Second, we divided patients into anhedonic and nonanhedonic MDD to explore the differences in cytokines, CRP, and cortisol between the two groups. Third, the relationships of serum cytokines, CRP, and cortisol with the severity of depression and anhedonia were examined in MDD. Finally, we combined these biological factors to explore the predictive value for distinguishing MDD from HC and distinguishing MDD with anhedonia from those without anhedonia. We hypothesized that anhedonic MDD has distinctive neuroendocrine-immune characteristics, and the combinations of the biological factors can be eligible predictors for MDD with anhedonia.

## Materials and methods

### Participants

All inpatients were recruited from the psychosomatics and psychiatry departments of Zhongda Hospital affiliated with Southeast University between January 2020 and February 2021. Experienced psychiatrists determined the clinical diagnoses of MDD based on the Structured Clinical Interview of DSM-5. The inclusion criteria were as follows: (1) aged from 18 to 65 years; (2) Hamilton Rating Scale for Depression-24 (HAMD-24) ≥20. Participants who met the following criteria were excluded: (1) serious physical illnesses (e.g., tumor, cardiac disease, organic brain diseases, hepatic disease, or renal disease); (2) other mental disorders (e.g., intellectual disability, bipolar disorder, or schizophrenia); (3) women in pregnancy or puerperium; (4) autoimmune diseases, acute and chronic infections, or allergies; (5) substance abuse/dependence, including drugs, alcohol, or tobacco; (6) use of anti-inflammatory drugs, probiotics, prebiotics, antibiotics, or corticosteroids in the past 4 weeks; and (7) received antidepressants or antipsychotics treatment in the past 4 weeks. At the same time, HCs who met the same exclusion criteria were recruited through advertisements. All HCs assessed HAMD-24 < 8 and the Snaith-Hamilton Pleasure Scale (SHAPS)≤5.

### Measures

We used a self-designed questionnaire to investigate the general situation of all participants, including age, sex, body mass index (BMI), years of education, marital status, episode times, and duration of illness.

The Chinese version of SHAPS was adopted to assess the severity of anhedonia. It consists of 14 self-rated items. Each item has four categories of response ranging from “strongly agree” to “strongly disagree,” where “strongly agree” or “equally agree” score is 0 and “disagree” or “strongly disagree” score is 1. The total score ranges from 0 to 14, with higher scores indicating more severe anhedonia (Liu et al., 2012). Total SHAPS scores > 5 were recognized as MDD with a significant anhedonic tone (Wu et al., 2020; Tang et al., 2021). The Chinese version of HAMD-24 was employed to assess depression severity in patients with MDD, which has demonstrated test-retest reliability and good internal consistency.

Blood samples (5 ml) were collected from the forearm veins of fasting participants at approximately 8 a.m. The blood was centrifuged to obtain serum at 3,000 revolutions per minute for 10 min at 4°C. The isolated serum was stored in a refrigerator at −80°C before analysis.

Serum levels of cytokines (TNF-α, IL-2, IL-4, IL-6, IL-10, IL-17A, and IFN-γ) were examined using commercially available flow cytofluorometric (FCM) kits (SaijiBiotech, Nanchang, Jiangxi, China); the levels of cortisol and CRP were detected using commercially available enzyme-linked immunosorbent assay (ELISA) kits (cortisol kit from R&D Systems, Mutlukent Mah, Arda Sk, USA; CRP kits from RayBiotech, Norcross, GA, USA). All blood parameters were tested following the manufacturer's instructions.

### Statistical analysis

SPSS version 22.0 (SPSS, Inc., Chicago, IL, USA) statistical software was used to perform the data analyses. Data were presented as the mean ± standard error of the mean (SEM). An independent sample *t*-test was taken to evaluate the discrepancy of the continuous variables (age, years of education, BMI, episode times, duration of illness, HAMD-24 score, SHAPS score, cytokines, CRP, and cortisol) between groups. The *chi-square* test was taken to analyze the discrepancy of categorical variables (sex and marital status) between groups. The correlations between variables (HAMD-24 score, SHAPS score, cytokines, CRP, and cortisol) were evaluated by Spearman correlation analysis. With cytokines, CRP, and cortisol entered, multiple stepwise regression analysis was taken to determine the independent factors that significantly affected SHAPS scores in MDD. Finally, the predictive values of cytokines, CRP, and cortisol were estimated using receiver operating characteristic (ROC) curve analysis. The sensitivity, specificity, and area under the curve (AUC) were calculated. All analyses were two-tailed, and the significance was set as *P* < 0.05.

## Results

### Characteristics of the participants

A total of 80 (95.2%) patients with MDD agreed to participate among 84 patients eligible for inclusion in this study. Patients with 14 MDD, however, did not complete the scales, did not draw blood samples, or volunteered to withdraw. Finally, 66 patients with MDD completed the study, with a response rate of 78.5%. We recruited 69 HCs, 66 of whom met the criteria for admission and completed psychological scale tests as well as blood examination. There were no significant differences in age, sex, BMI, years of education, and marital status between the two groups (all *P* > 0.05). In the MDD group, the episode times were 1.52 ± 0.12, the duration of illness was 30.58 ± 7.55 months, and the HAMD-24 score was 29.85 ± 1.00. Increased SHAPS score was shown in the MDD group than that in the HC group (*P* < 0.01) (Table 1).

**Table 1:** Demographic and clinical data of participants in MDD and HC.

	**MDD (*n* = 66)**	**HC (*n* = 66)**	***t*/*X^2^***	***P***
Age (year)	44.11 ± 1.83	42.92 ± 1.58	0.489	0.626
Gender (male/Female)	14/52	17/49	0.379	0.682
BMI	22.71 ± 0.40	23.57 ± 0.40	1.527	0.129
**Marital status**				
Single/widower/divorced (%)	17 (23.1%)	16 (25.4%)	0.040	0.841
Married (%)	49 (76.9%)	50 (74.6%)		
Years of education (years)	11.23 ± 0.46	12.62 ± 0.76	1.566	0.120
Duration of illness (months)	38.58 ± 7.55	–	–	–
Episode times	1.52 ± 0.12	–	–	–
HAMD-24	29.85 ± 1.00	–	–	–
SHAPS	6.72 ± 0.45	0.52 ± 0.11	13.302	<0.001

The data represent the mean ± SEM or N.

According to item 2 of the symptom criteria (A) (loss of interest or pleasure) of the DSM-5 diagnostic criteria for MDD, along with SHAPS scores >5 based on previous studies (Wu et al., 2020; Tang et al., 2021), 66 patients were classified as anhedonic (*n* = 29) or nonanhedonic MDD (*n* = 37). No significant differences were found in age, sex, BMI, marital status, years of education, duration of illness, and episode times between the two groups (all *P* > 0.05). Although the HAMD-24 score of anhedonic MDD was higher than that of nonanhedonic MDD, no statistically significant discrepancy was shown (*P* > 0.05) (Table 2).

**Table 2:** Demographic and clinical data of MDD patients with or without anhedonia.

	**With anhedonia (*n* = 29)**	**Without anhedonia (*n* = 37)**	***t/X^2^***	***P***
Age (year)	46.07 ± 2.80	42.57 ± 2.43	0.947	0.347
Gender (male/Female)	7/22	7/30	0.265	0.607
BMI	22.80 ± 0.57	22.64 ± 0.56	0.205	0.839
**Marital status**				
Single/widower/divorced (%)	8 (27.59%)	9 (24.32%)	0.090	0.764
Married (%)	21 (72.41%)	28 (75.68%)		
Years of education (years)	10.28 ± 0.75	11.97 ± 0.55	1.864	0.067
Duration of illness (months)	43.45 ± 15.69	27.65 ± 5.51	1.039	0.303
Episode times	1.62 ± 0.24	1.43 ± 0.10	0.785	0.436
HAMD-24	31.76 ± 1.47	28.35 ± 1.12	1.876	0.065
SHAPS	10.00 ± 0.44	3.87 ± 0.21	12.463	<0.001

The data represent the mean ± SEM or N.

### Comparisons of serum cytokines, CRP, and cortisol between MDD and HC

**Figure 1:**
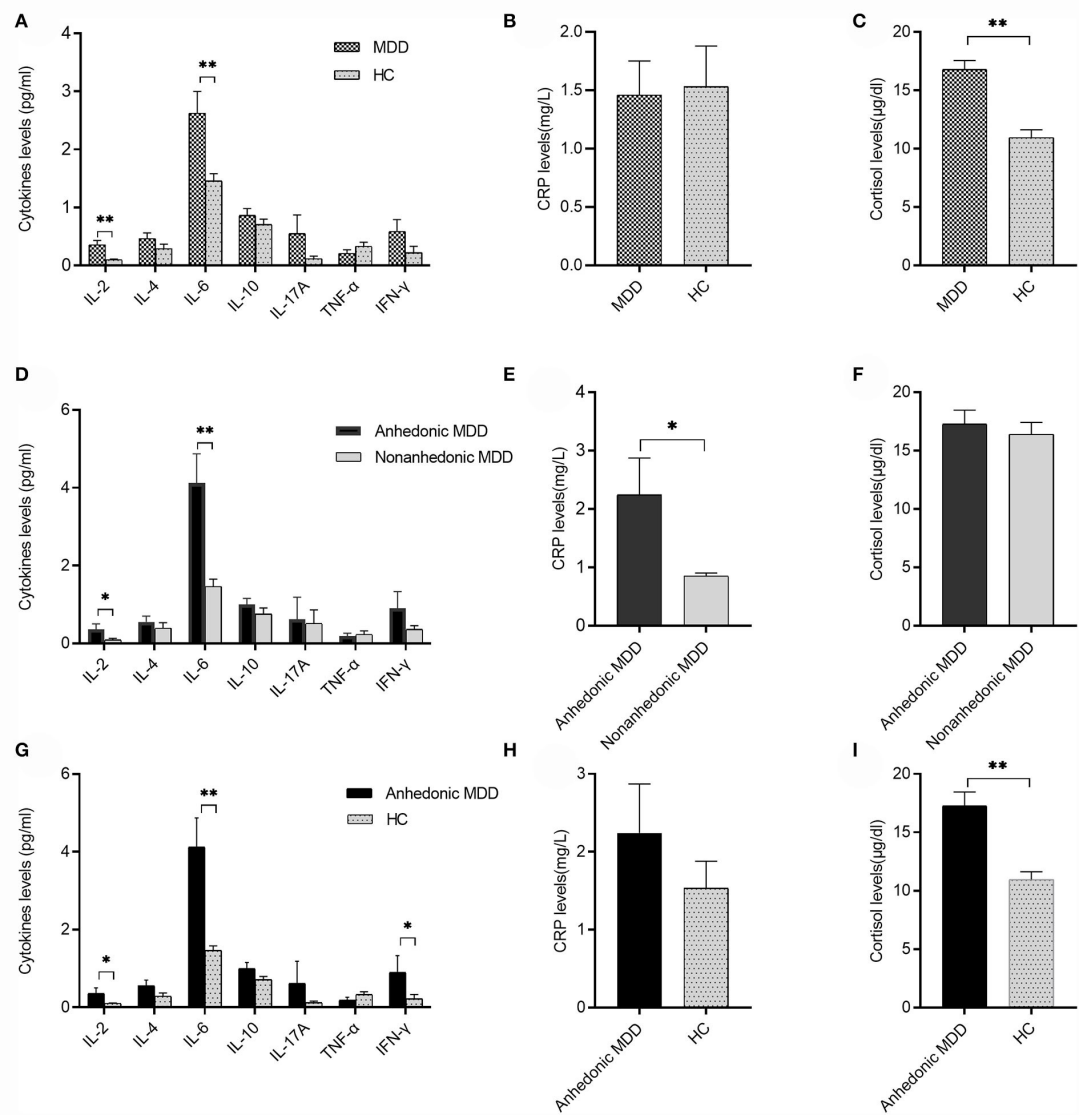
Serum cytokines, C-reactive protein (CRP), and cortisol levels between major depressive disorder (MDD) and healthy control (HC) or anhedonic and nonanhedonic MDD. **(A)** Serum levels of IL-2 and IL-6 in the MDD group were significantly higher than the HC group (*t* = 2.855, 2.922, respectively, both *P* < 0.01); **(B)** there was no significant difference in CRP levels between MDD and HC (*P* > 0.05). **(C)** Serum levels of cortisol in the MDD group were significantly higher than those in the HC group (*t* = 5.703, *P* < 0.001); **(D)** serum levels of IL-2 and IL-6 in anhedonic MDD were higher than those in nonanhedonic MDD (*t* = 2.025, 3.426 respectively, *P* < 0.05 or 0.01); **(E)** serum levels of CRP in anhedonic MDD were higher than those in nonanhedonic MDD (*t* = 2.476, *P* < 0.05); **(F)** no significant difference was found in serum cortisol levels between anhedonic and nonanhedonic MDD (*P* > 0.05); **(G)** serum levels of IL-2, IL-6, and INF-γ in anhedonic MDD were significantly higher than the HC group (*t* = 2.443, 3.493, 2.024, respectively, *P* < 0.05 or 0.01); **(H)** there was no significant difference in CRP levels between anhedonic MDD and HC (*P* > 0.05); **(I)** serum levels of cortisol in anhedonic MDD were significantly higher than those in HC (*t* = 4.891, *P* < 0.001). Mean ± SEM are shown. *P < 0.05, **P < 0.01.

Patients with MDD had elevated serum levels of IL-2 (0.36 ± 0.07 pg/ml vs. 0.10 ± 0.01 pg/ml, *t* = 2.855, *P* = 0.005), IL-6 (2.62 ± 0.38 pg/ml vs. 1.46 ± 0.12 pg/ml, *t* = 2.922, *P* = 0.004), and cortisol (16.79 ± 0.76 μg/dl vs. 10.95 ± 0.68 μg/dl, *t* = 5.703, *P* < 0.001) compared with the HC group (Figures 1A,C). However, patients with MDD did not differ from the HC group in serum levels of TNF-α, INF-γ, IL-4, IL-10, IL-17A, and CRP (all *P* > 0.05) (Figures 1A,B).

### Comparisons of serum cytokines, CRP, and cortisol between anhedonic and nonanhedonic MDD

Anhedonic MDD showed increased serum levels of IL-2 (0.36 ± 0.14 pg/ml vs. 0.09 ± 0.04 pg/ml, *t* = 2.025, *P* = 0.047), IL-6 (4.12 ± 0.75 pg/ml vs. 1.46 ± 0.19 pg/ml, *t* = 3.803, *P* < 0.001), and CRP (2.25 ± 0.64 mg/l vs. 0.85 ± 0.05 mg/l, *t* = 2.476, *P* = 0.016) compared with nonanhedonic MDD (Figures 1D,E). No significant difference was shown in serum levels of TNF-α, INF-γ, IL-4, IL-10, IL-17A, and cortisol between the two groups (all *P* > 0.05) (Figures 1D,F).

### Comparisons of serum cytokines, CRP, and cortisol between anhedonic MDD and HC

Anhedonic MDD showed increased serum levels of IL-2 (0.36 ± 0.14 pg/ml vs. 0.10 ± 0.01 pg/ml, *t* = 2.443, *P* = 0.021), IL-6 (4.12 ± 0.75 pg/ml vs. 1.46 ± 0.12 pg/ml, *t* = 3.493, *P* = 0.002), INF-γ (0.90 ± 0.43 pg/ml vs. 0.23 ± 0.11 pg/ml, *t* = 2.024, *P* = 0.046), and cortisol (17.28 ± 1.18 μg/dl vs. 10.95 ± 0.68 μg/dl, *t* = 4.891, *P* < 0.001) compared with the HC group (Figures 1G,I). No significant difference was shown in serum levels of TNF-α, IL-4, IL-10, IL-17A, and CRP between the two groups (all *P* > 0.05) (Figures 1G,H).

### Correlation of cytokines, CRP, Cortisol, and Depression or Anhedonia in MDD

**Figure 2:**
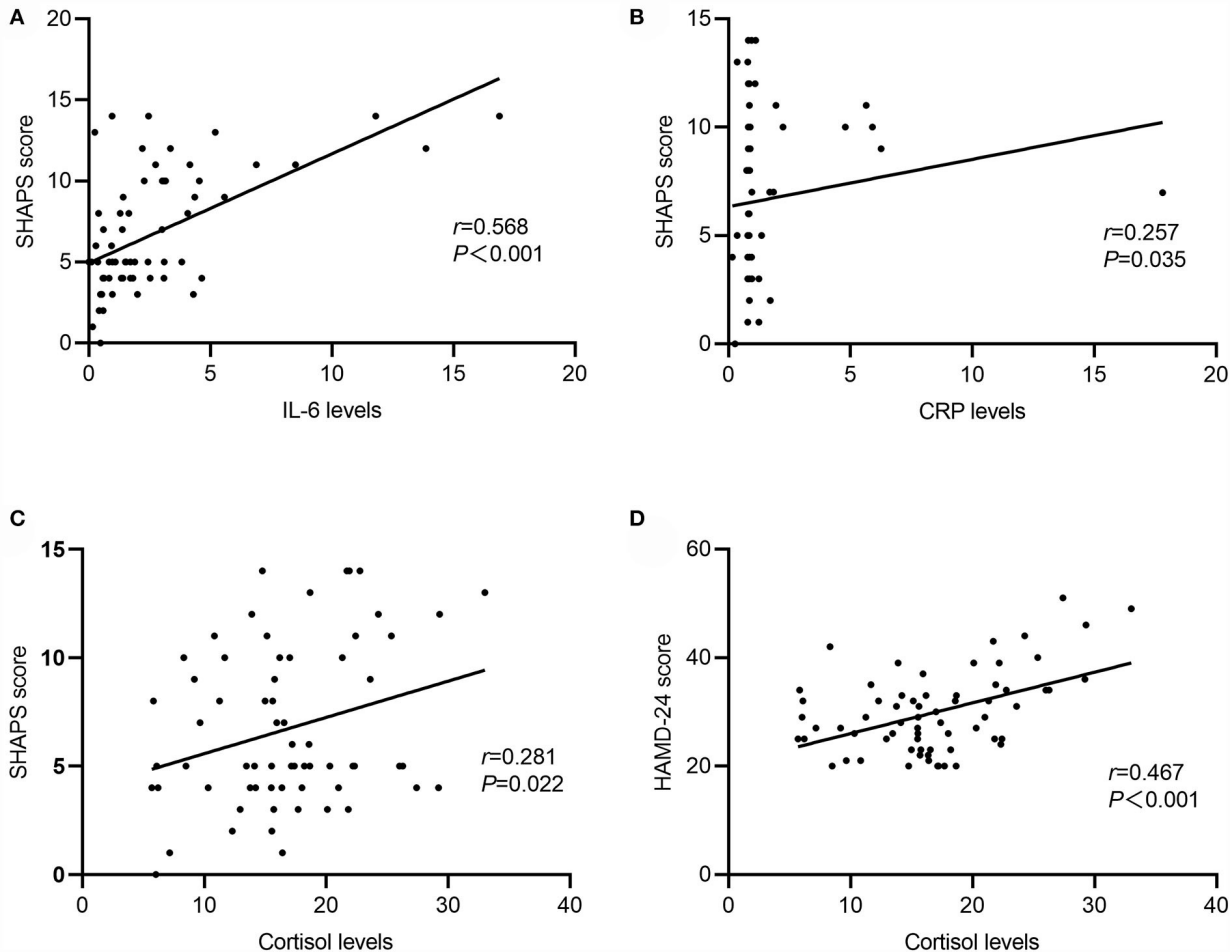
Correlation between serum cytokines, CRP, cortisol, Snaith-Hamilton Pleasure Scale (SHAPS), and Hamilton Rating Scale for Depression-24 (HAMD-24) in MDD. **(A)** IL-6 levels were positively associated with SHAPS score (*r* = 0.568, *P* < 0.001); **(B)** CRP levels were positively associated with SHAPS score (*r* = 0.257, *P* = 0.035); **(C)** cortisol levels positively associated with SHAPS score (*r* = 0.281, *P* = 0.022); **(D)** cortisol levels were positively associated with HAMD-24 score (*r* = −0.467, *P* < 0.001).

Correlation analyses exhibited that the SHAPS score was positively correlated with serum levels of IL-6 (*r* = 0.568, *P* < 0.001), CRP (*r* = 0.257, *P* = 0.035), and cortisol (*r* = 0.281, *P* = 0.022). Serum levels of IL-6 were positively related to serum levels of CRP (*r* = 0.267, *P* = 0.030). Additionally, a significant association was shown between the HAMD-24 total score and serum cortisol levels (*r* = 0.467, *P* < 0.001) (Figure 2).

The results of multiple stepwise regression analysis showed that the score of SHAPS was independently and positively related to serum levels of IL-6 (standardized β = 0.451, *P* < 0.001), CRP (standardized β = 0.220, *P* = 0.038), and cortisol (standardized β = 0.336, *P* = 0.002) (Table 3).

**Table 3:** Multiple stepwise regression analysis showing the variables independently associated with SHAPS score in MDD.

**Predictors**	**Unstandardized coefficients**		**Standardized coefficients**	***t***	***P***
	**B**	**SE**	**B**		
IL-6	0.349	0.080	0.451	4.369	<0.001
Cortisol	0.200	0.061	0.336	3.284	0.002
CRP	0.341	0.161	0.220	2.122	0.038

### Differentiating values of cytokines, CRP, and cortisol

**Figure 3:**
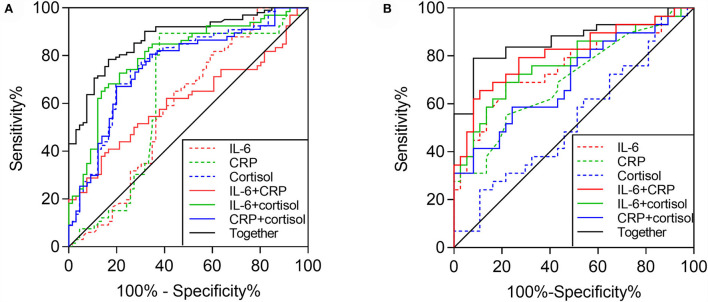
Area under the curves (AUCs) reflect the discriminating potential of different combinations of IL-6, CRP, and cortisol. **(A)** AUCs between MDD and HC (together: AUC = 0.873, *P* < 0.001; IL-6 + CRP: AUC = 0.611, *P* = 0.028; IL-6 + cortisol: AUC = 0.801, *P* < 0.001; CRP + cortisol: AUC = 0.756, *P* < 0.001; cortisol: AUC = 0.765, *P* < 0.001; CRP: AUC = 0.640, *P* = 0.006; and IL-6: AUC = 0.592, *P*=0.069); **(B)** AUCs between anhedonic and nonanhedonic MDD (together: AUC = 0.864, *P* < 0.001; IL-6 + CRP: AUC = 0.811, *P* < 0.001; IL-6 + cortisol: AUC = 0.722, *P* < 0.001; CRP + cortisol: AUC = 0.700, *P* = 0.005; IL-6: AUC = 0.754, *P* < 0.001; CRP: AUC = 0.697, *P* = 0.006; and cortisol: AUC = 0.532, *P* = 0.656).

The differentiated power was predicted using ROC curve analysis. IL-6, which differed significantly between groups and was positively associated with anhedonia, was chosen as a potential predictor along with CRP and cortisol. AUCs, specificity, and sensitivity of different combinations of IL-6, CRP, and cortisol for discriminating MDD and HC are shown in Table 4. The results showed that AUCs of single IL-6, CRP, and cortisol were 0.592, 0.640, and 0.765, respectively, which were not ideal distinguishing indicators. At the same time, the AUCs of the combinations of any two predictors (AUC = 0.611, 0.801, and 0.756, respectively) were higher than single indicators. The highest AUC was the combination of all three indicators (AUC = 0.873), with a sensitivity of 77.3% and specificity of 72.7% (Figure 3A).

**Table 4:** AUCs of differential combination of IL-6, CRP and cortisol for discriminating MDD and HC.

**Different combinations**	**AUC**	**Sensitivity**	**Specificity**
IL-6	0.592	54.5%	65.2%
CRP	0.640	89.4%	62.1%
Cortisol	0.765	74.2%	74.2%
IL-6 + CRP	0.611	83.3%	40.9%
IL-6 + cortisol	0.801	78.8%	72.7%
CRP + cortisol	0.756	72.7%	74.2%
IL-6 + CRP + cortisol	0.873	77.3%	72.7%

Table 5 presents the AUCs, specificity, and sensitivity of different combinations of IL-6, CRP, and cortisol for discriminating anhedonic and nonanhedonic MDD. AUCs of single IL-6, CRP, and cortisol were 0.754, 0.693, and 0.532, respectively. Similarly, AUCs of the combinations of any two indicators (AUC = 0.811, 0.772, and 0.700, respectively) were increased compared with single predictors. The maximal AUC combined these three indicators (AUC = 0.864), with a sensitivity of 79.3% and specificity of 72.9% (Figure 3B).

**Table 5:** AUCs of differential combination of IL-6, CRP and cortisol for discriminating MDD with/without anhedonia.

**Different combinations**	**AUC**	**Sensitivity**	**Specificity**
IL-6	0.754	70%	79.4%
CRP	0.697	55.2%	78.4%
Cortisol	0.532	24.1%	88.2%
IL-6 + CRP	0.811	65.5%	91.9%
IL-6 + cortisol	0.772	69.0%	78.4%
CRP + cortisol	0.700	58.4%	75.7%
IL-6 + CRP + cortisol	0.864	79.3%	72.9%

## Discussion

This study exhibited that serum levels of pro-inflammatory cytokines IL-2 and IL-6 were elevated in patients with MDD compared with healthy subjects, primarily in agreement with previous research (Eker et al., 2014; Ting et al., 2020). However, our results showed no significant difference in anti-inflammatory factors IL-4 and IL-10 and pro-inflammatory markers IL-17A, TNF-α, IFN-γ, and CRP. Although it is widely acknowledged that chronic low-level inflammatory responses play roles in the development of MDD (Köhler-Forsberg et al., 2017), there is no consensus on which inflammatory markers are abnormal compared with normal controls (Himmerich et al., 2019). Studies have shown elevated levels of CRP, IL-6, and TNF-α in MDD (Haapakoski et al., 2015; Köhler et al., 2017). This study found no increased TNF-α and CRP levels in patients with MDD, which is inconsistent with some other studies (Haapakoski et al., 2015; Jha et al., 2019). The divergence may be due to several confounding factors, including age, body weight, medication, heterogeneity of the disorder, and different subtypes of MDD (Tang et al., 2021). Interestingly, in the comparisons of subgroups, anhedonic MDD not only exhibited higher IL-2 and IL-6 levels but also higher CRP levels than nonanhedonic MDD. The findings extend the result of a previous study which found only elevated plasma levels of IL-6 in anhedonic MDD compared with those in nonanhedonic MDD (Tang et al., 2021). These elevated inflammatory markers suggest that anhedonic MDD may demonstrate more pronounced immune disturbance and present different immunological characteristics. Furthermore, elevated levels of IL-2, IL-6, CRP, and anhedonia were related to poor treatment response in MDD (Craske et al., 2016; Zhang et al., 2019; Mosiołek et al., 2021). In addition to increased serum IL-2 and IL-6 levels, elevated INF-γ levels were also found in the anhedonic MDD group compared with those in the HC group. One animal study showed that higher IFN-γ levels were correlated with anhedonia-like behavior in stressed rats (Géa et al., 2019). An association between anhedonic symptoms and INF-γ was also demonstrated in adolescents with MDD (Freed et al., 2019). Our study, combined with the two studies mentioned above, suggests that INF-γ may also be an important pro-inflammatory cytokine in immune-inflammatory pathways in patients with anhedonic MDD. These findings may provide a certain theoretical basis for exploring new treatment strategies such as anti-inflammatory interventions for anhedonic MDD.

In addition, we found that serum cortisol levels increased in patients with MDD compared with those in HCs. Higher cortisol levels are considered one of the important features of depression (Vreeburg et al., 2009; Nguyen et al., 2020). Consistent with previous studies, this study supports that hyperactivation of the HPA axis may be involved in the pathogenesis of MDD. As far as we know, this is the first study to measure cortisol in MDD using anhedonia as a grouping feature. However, we did not observe a significant difference between anhedonic MDD and nonanhedonic MDD. This study seems inconsistent with the previous studies in humans and animals (Fernandes et al., 2018; Cunningham et al., 2021), where higher cortisol was connected with more severe anhedonia. It may result from different methods, including the presence of laboratory stressors and groupings. The role of the HPA axis in the pathogenesis of anhedonia in MDD needs to be further studied.

In this study, serum CRP and IL-6 were positively associated with anhedonia, whereas cortisol levels were related to both severities of depression and anhedonia in MDD. No relationship was found between IL-6, CRP, and the severity of depression. The stepwise regression analysis ulteriorly determined that elevated CRP, IL-6, and cortisol levels were reciprocally and independently related to anhedonia. Prior studies have shown that anhedonia but not the depressive symptom is related to inflammatory markers, mainly CRP and IL-6 in MDD (Bekhbat et al., 2020; Kudinova et al., 2020; Majd et al., 2020; Rengasamy et al., 2021), which was consistent with our results suggesting the role of inflammation in patients with MDD with anhedonia. Similarly, another research in line with this study revealed that higher levels of anhedonia are positively associated with cortisol reactivity to stress (Cunningham et al., 2021). In addition, previous fMRI studies have demonstrated that brain reward circuitry in anhedonia is related to inflammation and cortisol levels in MDD (Felger et al., 2016; Harrison et al., 2016; Nguyen et al., 2020). For example, Felger et al. indicated that increased inflammation, especially CRP and IL-6 levels, were associated with reduced functional connections involved in brain reward circuits in MDD (Felger et al., 2016). Nguyen et al. (2020) found that elevated cortisol levels were related to reductions in the prefrontal network, a principal brain region involved in reward processing in MDD. The studies mentioned above further support our research suggesting that anhedonia is closely related to inflammation markers and the HPA axis, which may play a crucial part in the pathogenesis of MDD.

This study found that the distinguishing value of serum cortisol was relatively high compared with CRP and IL-6 between MDD and normal controls, which is consistent with previous studies (Jia et al., 2019), suggesting that cortisol is a relatively better distinguishing indicator in terms of single indicators. Nevertheless, the AUC of cortisol was only 0.745 in this study, presenting adequate clinical differential capacity. As we all know, there has been a lack of effective biomarkers for diagnosing depression due to the complex pathogenesis (Krajčíková et al., 2021), and particularly single biomarkers were not satisfactory (Pratt and Stapelberg, 2018). Therefore, we further explored the distinguishing values of different combinations of IL-6, CRP, and cortisol. The combination of three factors had the best distinguishing value, achieving AUC 0.873 with a sensitivity of 77.3% and specificity of 72.7%, which has practical clinical significance. This study suggests that the combination of multiple biomarkers is likely to enhance the discriminative power, just as some previous studies have mentioned (van Buel et al., 2019; Jentsch et al., 2020). With regard to the distinguishing values of the three factors between anhedonic and nonanhedonic MDD, we found that the differentiating effect of serum IL-6 was higher than CRP and cortisol, indicating that serum IL-6 might be a comparatively better predictive indicator as single markers to distinguish anhedonic MDD from nonanhedonic MDD. However, the AUC of IL-6 was only 0.754, suggesting that the clinical differentiation value was not particularly good. Similarly, we found the combination of IL-6, CRP, and achieving AUC 0.864 with a sensitivity of 79.3% and specificity of 72.9%. This is the first study to utilize biomarkers for distinguishing MDD with or without anhedonia. This study suggests that the combination of IL-6, CRP, and cortisol can be an excellent indicator to differentiate MDD with or without anhedonia, which might help clinicians identify patients with MDD with anhedonia early, thus developing more appropriate treatment strategies to achieve better outcomes.

There were several limitations in our study. First, the relatively small sample might lead to negative results of cytokines, CRP, and cortisol between groups. Second, not all the patients with MDD were drug-naïve. Some of them received antidepressants in the past but stopped taking them 4 weeks before enrollment, which might partly influence the levels of cytokines, CRP, and cortisol. Third, due to the cross-sectional design of this study, we could not conclude causality on the relationships between anhedonia, cortisol cytokines, and CRP in patients with MDD. Fourth, the limited number of cytokines and the only morning cortisol levels were measured. Moreover, the gut microbiota was not involved in this study. Recently, the gut microbiota has been at the forefront of depression research, and the relationship between altered intestinal microbiota, elevated inflammation markers, and activated HPA axis in MDD has received much attention (Frankiensztajn et al., 2020; Mason et al., 2020). The combination of intestinal microbiota, immunity, and HPA axis will be more valuable for the exploration of the pathogenesis of depression. Therefore, future longitudinal studies involving altered gut microbiota, more inflammatory markers, more endocrine indicators reflecting HPA axis function, and larger sample sizes are needed to explore the mechanism of depression in the microbial-gut-brain axis in MDD.

## Conclusion

This study suggests that increased IL-2, IL-6, and CRP are involved in the pathophysiology of MDD with anhedonia. Cortisol levels are related to both severity of depression and anhedonia, whereas the levels of IL-6 and CRP are merely related to anhedonia, which suggests that patients with anhedonic MDD have unique neuroendocrine-immune characteristics. The combination of IL-6, CRP, and cortisol might be served as an early differentiating marker to distinguish MDD with anhedonia from those without anhedonia.

## Data availability statement

The raw data supporting the conclusions of this article will be made available by the authors, without undue reservation.

## Ethics statement

The studies involving human participants were reviewed and approved by the Ethics Committee of the Affiliated ZhongDa Hospital of Southeast University. The patients/participants provided their written informed consent to participate in this study.

## Author contributions

YYua designed this study and helped with manuscript revision. YL participated in scale evaluation, blood sample collection, data analysis, and writing. YYue, SC, ZX, GC, and ZZ evaluated the scales, collected blood samples, and interpreted the findings. WJ participated in the interpretation of findings, writing, and revision of the manuscript. LT participated in scale evaluation and blood sample collection. All authors have read and approved the final manuscript.

## Funding

This study was supported by the Medical Innovation Team of Jiangsu Province (2017ZXKJQW10).

## Conflict of interest

The authors declare that the research was conducted in the absence of any commercial or financial relationships that could be construed as a potential conflict of interest.

## Publisher's note

All claims expressed in this article are solely those of the authors and do not necessarily represent those of their affiliated organizations, or those of the publisher, the editors and the reviewers. Any product that may be evaluated in this article, or claim that may be made by its manufacturer, is not guaranteed or endorsed by the publisher.
